# Overcoming the *H‐NO*_x_* Coupling Bottleneck via Palladium‐Enabled Relay Catalysis on CuCo_2_O_4_ Toward Ampere‐Level Nitrate‐to‐Ammonia

**DOI:** 10.1002/advs.78576

**Published:** 2026-10-29

**Authors:** Yuhuan Cui, Yingying Xu, Jingyao Guo, Yao Liu, Yuxuan Li, Zixuan Feng, Yitong Zhou, Yongjun Gao, Zhili Wang

**Affiliations:** ^1^ Hebei Key Laboratory of Analytical Science and Technology, and College of Chemistry and Materials Science Hebei University Baoding People's Republic of China; ^2^ Key Laboratory of Automobile Materials Ministry of Education and School of Materials Science and Engineering Jilin University Changchun People's Republic of China; ^3^ Institutes of Physical Science and Information Technology Anhui University Hefei People's Republic of China

**Keywords:** CuCo_2_O_4_, *H‐NO*_x_* coupling, nitrate reduction to ammonia, Pd modification, relay catalysis

## Abstract

Electrocatalytic nitrate reduction reaction (NO_3_RR) represents a sustainable route for both ammonia (NH_3_) production and nitrate‐contaminated water remediation, yet its practical application is crippled by the kinetically sluggish multi‐electron/proton transfer and insufficient targeted active hydrogen (*H) supply. Herein, a Pd‐doped CuCo_2_O_4_ (Pd‐CuCo_2_O_4_) hollow spiny microsphere catalyst is designed to tackle this challenge via a synergistic relay catalysis mechanism coupled with rapid *H replenishment. Kinetics studies and theoretical calculations collectively demonstrate a clear tandem pathway for NH_3_ synthesis: NO_3_^−^ is preferentially reduced to NO_2_^−^ on Cu sites, and then migrates to neighboring Co sites for further conversion to NH_3_. Crucially, the incorporated Pd dopants promote NO_3_^−^ adsorption on Cu sites and *H generation on Co sites precisely. The efficient *H supply and co‐adsorption for intermediates eliminates the intrinsic kinetic bottleneck of poor *H‐NO*_x_* coupling, ensuring the tandem catalysis proceeds smoothly without interruption. Benefiting from the *H‐spillover boosted relay mechanism, an ultra‐high current density over −2.0 A cm^−2^ and a remarkable NH_3_ yield rate of 100.43 mg h^−1^ cm^−2^ are achieved. This study underscores the synergistic interplay among the three active sites and provides a blueprint for designing high‐rate, practical NO_3_RR electrocatalysts.

## Graphical abstract

Pd‐doped hollow spiny CuCo_2_O_4_ microspheres deliver ampere‐level electrocatalytic nitrate to ammonia. Cu and Co sites undergo relay catalysis for sequential nitrate reduction, while Pd accelerates water dissociation to supply abundant active hydrogen (*H). This synergistic design circumvents the critical *H‐NO_x_ coupling bottleneck and enables high ammonia yield of 100.43 mg h^−1^ cm^−2^, showing great potential in practical applications.

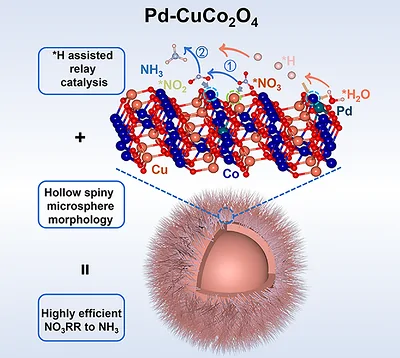

## 1 Introduction

Ammonia (NH_3_) serves as a cornerstone in modern agriculture [1, 2], as well as a promising carbon‐free hydrogen energy carrier [3, 4]. Up to now, Haber‐Bosch process for NH_3_ synthesis is predominant for the cost of high energy input and a large amount of greenhouse gas emissions [5, 6], thus necessitating the exploration of alternative and sustainable NH_3_ synthesis route. Electrocatalytic N‐containing substances such as nitrogen (N_2_), nitrogen oxides (NO*_x_*) and nitrates (NO*_x_* ^−^) to NH_3_ has been considered as a promising strategy [7, 8]. Thereinto, nitrate reduction reaction (NO_3_RR) could convert excessive nitrate pollutants in water into high‐value‐added NH_3_, which not only generates NH_3_ through decarbonization trend but also tackles environmental contamination [9, 10]. However, notable challenges including low NH_3_ yield and unsatisfactory Faradaic efficiency (FE) still hinder its practical application. Recent research has focused on accelerating reaction kinetics by designing efficient catalysts. For instance, Chen et al. constructed atomically dispersed Ru─Cu dual‐atom catalyst through a pulsed discharge method [11]. The tailored asymmetric structure of the catalyst achieves a significant improvement in FE (95.7%). Shan et al. reported a polyaniline‐based molecular electrode with a Cu catalyst in which polyaniline facilitates the protonation of key intermediates, thereby circumventing the limitations of the Cu catalyst in the efficiency‐limiting proton transfer step, significantly accelerating reaction kinetics and inhibiting hydrogen evolution reaction (HER) [12]. Despite recent progress have been made, the difficulties in NO_3_^−^ adsorption, the deeper reduction of intermediates along with the mismatch between active hydrogen species (*H) and intermediates are still urgently needed to be solved.

It is recognized that the nitrite (NO_2_^−^) is the key intermediate and thus NO_3_RR can be degraded into two main stages summarized as NO_3_^−^ → NO_2_^−^ and NO_2_^−^ → NH_3_ [13, 14]. Previous studies have demonstrated that Cu‐based catalysts could effectively activate NO_3_^−^ → NO_2_^−^ while the deeper reduction process to NH_3_ is hindered [15, 16]. On this basis, introducing Co sites with excellent NO_2_^−^ adsorption ability [16, 17] to construct Cu─Co relay active sites holds great potential in overcoming the limitations of the multi‐stages NO_3_RR [18, 19]. For example, Seh and co‐workers constructed spin‐related Cu─Co pair on high‐entropy oxides and found that synergistic effect of Cu─Co pair is beneficial to NH_3_ synthesis [18]. Nevertheless, inadequate *H supply arising from poor H_2_O adsorption and dissociation hinders the hydrogenation steps on Co sites [20]. Given this, boosting *H generation at Co sites and enabling its deep coupling with NO_2_^−^ are decisive factors in enhancing the overall NO_3_RR kinetics.

Inspired by the above‐mentioned key factors, we developed a novel Pd doped CuCo_2_O_4_ (marked as Pd‐CuCo_2_O_4_) catalyst for NO_3_RR via hydrothermal and calcination process. Experimental and density functional theory (DFT) results reveal that Cu and Co sites in CuCo_2_O_4_ accomplish the relay reactions from NO_3_^−^ → NO_2_^−^ and NO_2_^−^ → NH_3_, respectively. Notably, the incorporation of Pd facilitates NO_3_^−^ adsorption on Cu sites as well as H_2_O adsorption and *H generation on unoccupied Co sites. The localized *H replenishment enables Co to serve as a highly efficient bi‐functional coupling center. The co‐adsorption of *H and NO_2_^−^ on Co sites promotes the hydrogenation steps, ensuring that the NO_2_^–^ intermediate promptly consumed rather than accumulating or desorbing. In addition, the unique hollow spiny sphere morphology of Pd‐CuCo_2_O_4_ catalyst ensures the enough active sites and deep reduction process. Benefiting from the Pd‐assisted Cu─Co relay catalysis mechanism with optimized hydrogenation process, the Pd‐CuCo_2_O_4_ catalyst exhibits the NH_3_ yield rate of 100.43 mg h^−1^ cm^−2^ under ampere‐level current density, surpassing most recently reported catalysts. This work opens an avenue for designing synergistic catalyst and accelerates the practical applications of NO_3_RR.

## 2 Results and Discussion

### 2.1 Synthesis and Characterizations of Pd‐CuCo_2_O_4_ Catalyst

As shown in Figure S1, the Pd‐CuCo_2_O_4_ catalyst was prepared by a hydrothermal method using Cu(NO_3_)_2_·3H_2_O, Co(NO_3_)_2_·6H_2_O and Na_2_PdCl_4_ as the metal sources in the presence of urea and NH_4_F, followed by a calcination process in air. The scanning electron microscope (SEM) image shows that spiny nanowires are grown on hollow microspheres with a diameter of about 9 µm in the Pd‐CuCo_2_O_4_ catalyst (Figure 1a), which is shaped like a chestnut spiny shell (inset of Figure 1a). Transmission electron microscope (TEM) image further indicates that the spiny nanowires possess a porous structure, which enlarges the specific surface area and enables full exposure of active sites (Figure 1b). The outer spike‐like structure could effectively capture reactants, while the hollow structure could confine the intermediates for further reduction reaction to obtain NH_3_ product. Consequently, this unique hollow structure not only provides a large surface area with enough active sites but also enhances electrocatalytic activity through facilitating reactants adsorption and intermediates reduction. The corresponding energy dispersive spectroscopy (EDS) elemental mapping images in Figure 1d show the homogeneous distribution of Co, Cu, Pd, and O elements across the Pd‐CuCo_2_O_4_ nanowires. For comparison, to identify the specific role of Cu and Co sites, Pd‐CuO*_x_* and Pd‐Co_3_O_4_ counterparts were synthesized by the same method except that only equal total molar amount of Cu(NO_3_)_2_·3H_2_O or Co(NO_3_)_2_·6H_2_O was added during the preparation processes, respectively. It is worth noting that Pd‐CuO*_x_* catalyst exhibits a microsphere morphology composed of stacked nanosheets (Figure S2a) while Pd‐Co_3_O_4_ catalyst consists of spiky nanosheets (Figure S2b). As shown in Figure S2c, CuCo_2_O_4_ counterpart was synthesized to verify the effect of Pd doping through the same method except Na_2_PdCl_4_ was absent, and achieved the similar morphology to Pd‐CuCo_2_O_4_, indicating that the introduction of Pd shows negligible influence on the formation of spiny hollow microspheres. Figure 1c shows the X‐ray diffraction (XRD) patterns of Pd‐CuCo_2_O_4_ catalyst, exhibiting distinct peaks at 31.08°, 36.62°, 38.31°, 58.96° and 64.80°, which are corresponded to Cu_0.95_Co_2.05_O_4_ (JCPDS#78‐2177) [21]. No metallic Pd peaks were detected, indicating the low content and highly dispersed Pd species in Pd‐CuCo_2_O_4_ catalyst. Inductively coupled plasma optical emission spectroscopy (ICP‐OES) result confirms that the Pd content is about 1.7 at%. Figure 1e depicts the high‐resolution TEM (HRTEM) image, where the interplanar spacing of 0.245 nm corresponds to (311) plane of CuCo_2_O_4_ [22], further confirm ing the formation CuCo_2_O_4_, which is consistent with the XRD result. In addition, diffraction peaks at 44.51°, 51.85° and 76.37° are corresponded to the metallic Ni substrate (JCPDS#04‐0850) [23]. As shown in Figure S3, the XRD pattern of the control groups were also tested. The CuCo_2_O_4_ catalyst exhibits the similar diffraction peaks with Pd‐CuCo_2_O_4_. The Pd‐Co_3_O_4_ catalyst shows peaks at 31.27°, 36.85°, 59.36°, and 65.24°, which are indexed to Co_3_O_4_ phase (JCPDS#42‐1467) [24, 25]. Whereas Pd‐CuO*_x_* consist of CuO (JCPDS#80‐0076) with the diffraction peaks at 35.54°, 38.64° and 48.85° [26] as well as Cu_2_O (JCPDS#99‐0041) locating at 36.42°, 42.31° and 61.37° [27].

**FIGURE 1:**
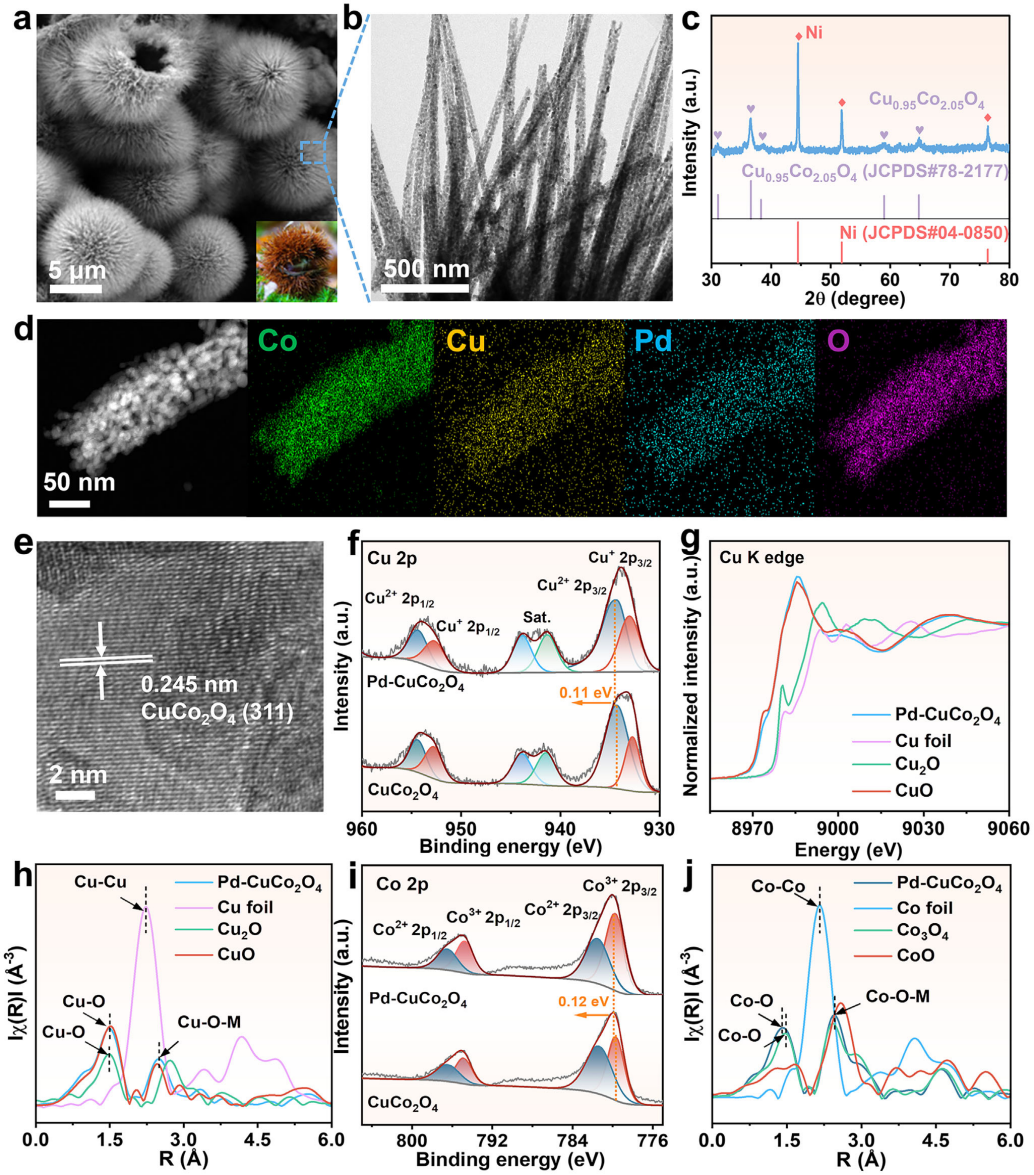
(a) SEM image, (b) TEM image, (c) XRD pattern, (d) TEM‐EDS mapping image, and (e) HAADF‐HRTEM image of Pd‐CuCo_2_O_4_ catalyst. (f) Cu 2p XPS spectra of Pd‐CuCo_2_O_4_ and CuCo_2_O_4_ catalysts. (g) Normalized Cu K‐edge XANES spectra and (h) Cu K‐edge FT‐EXAFS spectra of Pd‐CuCo_2_O_4_ catalysts along with the reference samples. (i) Co 2p XPS spectra of Pd‐CuCo_2_O_4_ and CuCo_2_O_4_ catalysts. (j) Co K‐edge FT‐EXAFS spectra of Pd‐CuCo_2_O_4_ catalysts along with the reference samples.

X‐ray photoelectron spectroscopy (XPS) survey spectra were used to analyze the elemental compositions and valence states of the as‐prepared catalysts. The Cu 2p XPS spectra of Pd‐CuCo_2_O_4_ and CuCo_2_O_4_ confirm the coexistence of Cu^2+^ and Cu^+^ species (Figure 1f). The binding energies at 934.35 and 954.35 eV assign to Cu^2+^ 2p_3/2_ and Cu^2+^ 2p_1/2_ for Pd‐CuCo_2_O_4_, respectively [28]. Compared with CuCo_2_O_4_, a positive binding energy shift of 0.11 eV in Pd‐CuCo_2_O_4_ is observed. In addition, Cu LMM Auger spectra of Pd‐CuCo_2_O_4_ and CuCo_2_O_4_ catalysts were also analyzed (Figure S4a). The Cu LMM peak of Pd‐CuCo_2_O_4_ shows a 0.60 eV positive shift in binding energy compared with CuCo_2_O_4_. The modified Auger parameter (*α*') was calculated and 0.49 eV reduction in α' upon Pd doping verifies the electron deficiency at Cu sites [28]. The Cu K‐edge X‐ray absorption near edge structure (XANES) demonstrates that Cu in Pd‐CuCo_2_O_4_ shows an average oxidation state close to +2, further confirming electron deficiency at Cu sites (Figure 1g). The Fourier‐transform extended X‐ray absorption fine structure (FT‐EXAFS) data further confirms Cu─O local coordination in the oxide lattice and excludes the metallic Cu species (Figure 1h). The above results mutually indicate that Pd doping induces electron loss of Cu sites. On the other hand, in the Co 2p XPS spectrum of Pd‐CuCo_2_O_4_, binding energies at 779.68 eV and 794.69 eV correspond to the Co^3+^ 2p_3/2_ and Co^3+^ 2p_1/2_ orbitals, respectively (Figure 1i) [29]. Compared to CuCo_2_O_4_, the Co binding energies in Pd‐CuCo_2_O_4_ shift positively by 0.12 eV, also manifesting the electronic modification through Pd doping effect. The O 1s spectrum can be divided into three peaks at about 529.80, 531.42, and 532.85 eV, corresponding to lattice oxygen (O_L_), oxygen vacancy (O_A_), and adsorbed moisture (O_W_), respectively (Figure S4b) [30]. In addition, the high‐resolution Pd 3d XPS spectrum confirms that Pd is dominantly stabilized as Pd^2+^ via Pd─O bonding (Figure S4c) [31]. According to the XPS results, the surface Pd content obtained from XPS is 1.2 at%, which is close to that of ICP‐OES. In addition, the Cu K‐edge and Co K‐edge FT‐EXAFS results reveal that the dominant peaks at ≈1.5 Å and ≈2.7 Å correspond to Cu─O and Co─O bonds, respectively (Figure 1h,j). Compared with the reference samples, the slight shift of Cu─O and Co─O peaks suggests the altered local coordination environment of Cu and Co in Pd‐CuCo_2_O_4_, which may arise from the possible formation of Cu─O─Pd/Co or Co─O─Pd/Cu bonds.

### 2.2 Electrochemical NO_3_RR Performance

Electrochemical NO_3_RR of Pd‐CuCo_2_O_4_ was conducted in an H‐type cell separated by Nafion 211 proton exchange membrane with a three‐electrode system [32]. Linear sweep voltammetry (LSV) curves were recorded for the four catalysts in 1.0 m KOH electrolyte with and without 0.1 m NO_3_^−^ to reveal the NO_3_RR responses. As shown in Figure 2a, Pd‐CuCo_2_O_4_ exhibits higher current density than CuCo_2_O_4_ in 1.0 m KOH electrolyte, suggesting Pd doping is beneficial for H_2_O dissociation and *H generation. After adding 0.1 m NO_3_^−^ into the electrolyte, the current densities show sharp increase in all the samples, indicating the occurrence of NO_3_RR on these Cu/Co‐based catalysts. Thereinto, Pd‐CuCo_2_O_4_ exhibits the highest current density under the same potential and reaches over −2.0 A cm^−2^ at −0.55 V (vs RHE), demonstrating its superior electrocatalytic activity by constructing Pd doped Cu─Co tandem sites. In addition, Pd‐CuCo_2_O_4_ obtains the onset potential (defined at −1.0 mA cm^−2^) of 0.39 V (vs RHE), which is higher than that of CuCo_2_O_4_ (0.36 V vs RHE), Pd‐Co_3_O_4_ (0.32 V vs RHE) and Pd─CuO*_x_* (0.36 V vs RHE), further confirming its outstanding NO_3_RR response (Figure S5). On this basis, chronoamperometry measurements for the four catalysts were conducted under various potentials ranging from 0 to −0.5 V (vs RHE) for 1.0 h (Figures S6–S9a). After electrolysis, the NH_3_ product in the electrolyte was quantified by ultraviolet–visible (UV–Vis) spectrophotometry (Figures S6–S9b) with standard equations determined by calibration curves (Figure S10). As demonstrated in Figure 2b, Pd‐CuCo_2_O_4_ exhibits the highest NH_3_ yield rate at each potential among all the catalysts and the value continues to increase with the potential shifts negatively, showing the maximum of 100.43 mg h^−1^ cm^−2^ at −0.5 V (vs RHE), which is almost twice that of Pd‐Co_3_O_4_ (47.25 mg h^−1^ cm^−2^) and Pd‐CuO*_x_* (53.72 mg h^−1^ cm^−2^), respectively, and surpassing most of the recently reported catalysts (Figure 2d and Table S2). Figure 2c displays the FE of NH_3_ (FE_NH3_) of the four catalysts at different potentials. Similarly, higher FE_NH3_ values were obtained with Pd‐CuCo_2_O_4_ catalyst at each potential compared to those single Cu/Co based control groups. In addition, it can be seen that the FE_NH3_ increases with the potential shifts negatively and reaches its highest value of 90.05% at −0.4 V (vs RHE) and then decreases at −0.5 V (vs RHE). This trade‐off arises because a more negative potential further accelerates NO_3_RR and increases the absolute NH_3_ production rate, yet simultaneously makes HER kinetically more favorable. Competition for electrons and adsorbed *H then leads to a decline in FE_NH3_. Besides, it can be clearly observed that after incorporating Pd into CuCo_2_O_4_, both the NH_3_ yield rate and FE_NH3_ improved dramatically compared to the CuCo_2_O_4_ counterpart. The pronounced performance gap indicates that Pd doping exerts a critical influence on the reaction pathway, warranting a closer examination of the underlying catalytic processes. To shed light on the nitrogen source of the NH_3_ product, isotope labeling experiments with quantitative ^1^H NMR analysis were conducted using K^14^NO_3_/K^15^NO_3_ as the reagents, respectively. As shown in Figure 2e, doublet peaks of ^15^NH_4_^+^ could be observed when using K^15^NO_3_ as the reagent, matching the result of ^15^NH_4_Cl standard curve. Similarly, triplet peaks of ^14^NH_4_^+^ are detected when K^14^NO_3_ was used as reagents [33]. However, no NH_4_^+^ signal appears when only KOH exists in the electrolyte, confirming that the produced NH_3_ originates exclusively from NO_3_RR rather than other contaminations. On this basis, the FE_NH3_ and NH_3_ yield rate were calculated by ^1^H NMR by establishing standard equation and the results are in good agreement with the data obtained by UV–Vis method (Figure S11), confirming the reliability of the quantitative methods.

**FIGURE 2:**
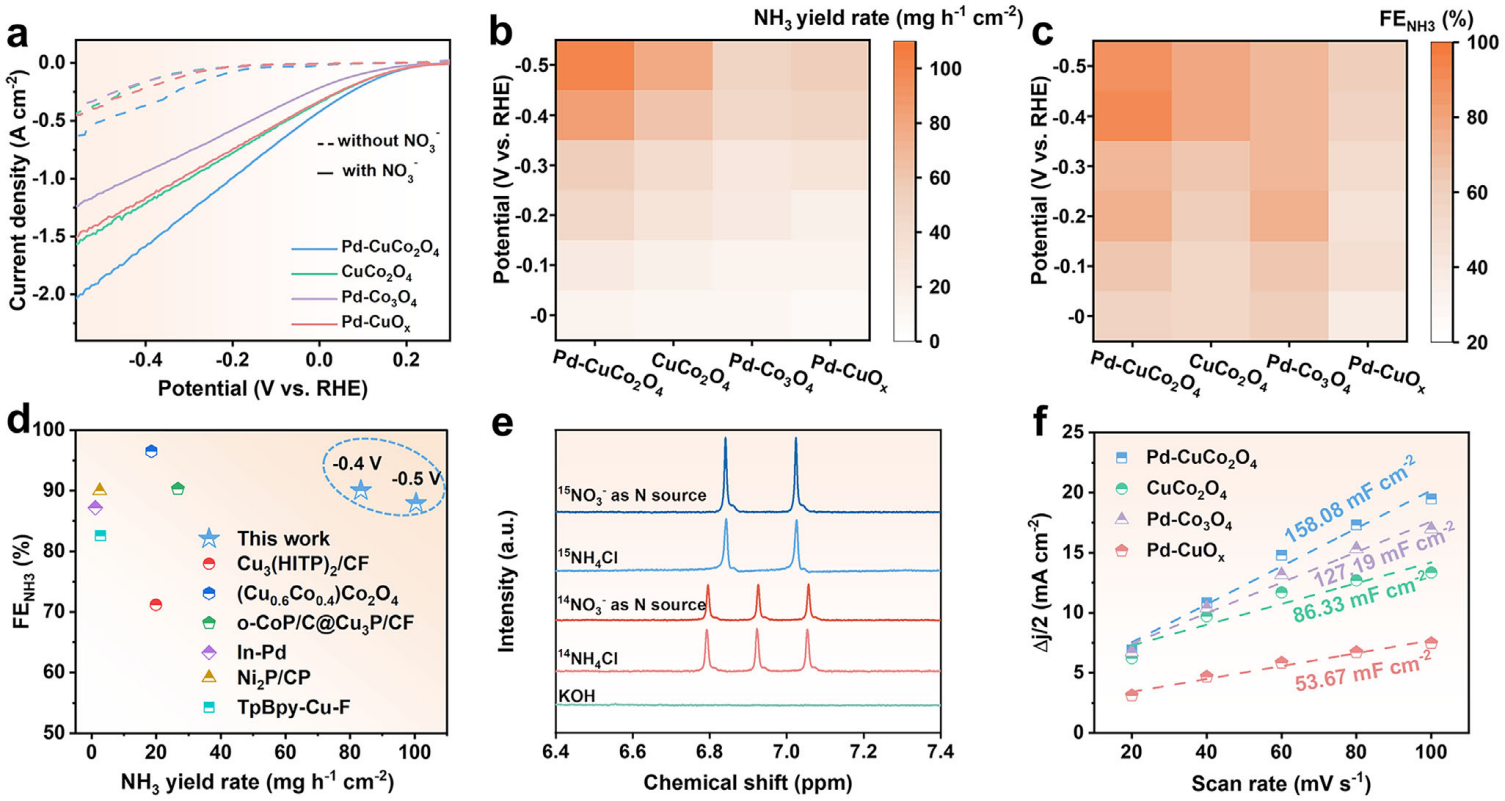
(a) LSV curves of Pd‐CuCo_2_O_4_, CuCo_2_O_4_, Pd‐Co_3_O_4_ and Pd‐CuO*_x_* catalysts in 1.0 m KOH + 0.1 m NO_3_^−^. (b) NH_3_ yield rate and (c) FE_NH3_ of Pd‐CuCo_2_O_4_, CuCo_2_O_4_, Pd‐Co_3_O_4_ and Pd‐CuO*_x_* catalysts at different potentials. (d) Comparison of NH_3_ yield rate and FE_NH3_ of Pd‐CuCo_2_O_4_ catalyst with other recently reported catalysts. (e) ^1^H NMR spectra from NO_3_RR at −0.4 V (vs RHE) using ^14^NO_3_^−^ or ^15^NO_3_^−^ as the reagents over Pd‐CuCo_2_O_4_ catalyst compared to the standard ^14^NH_4_^+^ and ^15^NH_4_^+^. (f) The *C*_dl_ of Pd‐CuCo_2_O_4_, CuCo_2_O_4_, Pd‐Co_3_O_4_ and Pd‐CuO*_x_* catalysts.

To better understand the origin of high NO_3_RR properties of Pd‐CuCo_2_O_4_, the electrochemically active surface area (ECSA) of each catalyst was determined by double‐layer capacitance (*C*_dl_) method (Figure S12) [34]. As displayed in Figure 2f, the *C*_dl_ value of Pd‐CuCo_2_O_4_ is 158.08 mF cm^−2^, which is higher than that of CuCo_2_O_4_ (86.33 mF cm^−2^), Pd‐CuO*_x_* (53.67 mF cm^−2^) and Pd‐Co_3_O_4_ (127.19 mF cm^−2^). Accordingly, the largest ECSA was obtained in Pd‐CuCo_2_O_4_ catalyst because of its high *C*_dl_, indicating that more active sites are available for electrocatalytic NO_3_RR. The electrochemical impedance spectroscope (EIS) Nyquist plots in Figure S13 offer information regarding the charge transfer resistance (*R*_ct_). Thereinto, Pd‐CuCo_2_O_4_ shows the lowest *R*_ct_ (7.20 Ω) among all the catalysts, indicating the fastest charge transfer kinetics, which is highly consistent with its superior electrocatalytic performance. In contrast, CuCo_2_O_4_ counterpart without Pd doping owns the largest *R*_ct_ of 11.96 Ω, suggesting the importance of Pd in accelerating electron transfer. On this foundation, Pd‐CuCo_2_O_4_ with different Pd contents were synthesized by modulating the Na_2_PdCl_4_ dosage to study the influence of Pd doping ratio. The atomic ratio of Pd was determined to be 0.9 at% and 2.8 at% by ICP‐OES (Table S1), and the corresponding catalysts were denoted as L‐Pd‐CuCo_2_O_4_ (low Pd content) and H‐Pd‐CuCo_2_O_4_ (high Pd content), respectively. SEM images indicate that adjusting the Pd content exerts barely any influence on the morphology of the catalyst (Figure S14). In addition, no Pd‐related phases were detected in the XRD patterns (Figure S15), suggesting that Pd possesses a low loading content with high dispersion and exists in a doped form in CuCo_2_O_4_. On this basis, NO_3_RR performances of L‐Pd‐CuCo_2_O_4_ and H‐Pd‐CuCo_2_O_4_ were tested through chronoamperometry and UV–Vis measurements (Figure S16a–d) and the comparison results were exhibited in Figure S16e,f. It can be observed that Pd‐CuCo_2_O_4_ with moderate Pd content (1.7 at%) exhibits higher NH_3_ yield rate and FE_NH3_ especially under high overpotentials. To understand such performance variation, XPS analysis was further conducted to reveal the electronic structure. It can be noted that the high‑resolution Cu 2p_3/2_ and Co 2p_3/2_ XPS spectra gradually shift to higher binding energies upon increasing Pd doping content, indicating continuous charge transfer from Cu/Co to Pd and an increased degree of electron deficiency for Cu/Co sites (Figure S17). Therefore, it can be inferred that the electronic modulation originating from Pd introduction has an optimal threshold under moderate Pd doping, which induces appropriate electron deficiency at Cu/Co sites to optimize the adsorption of intermediates and suppress the HER side reaction. However, excessive Pd doping triggers severe electron depletion of Cu/Co sites and strengthens H_2_ evolution activity, leading to lower NH_3_ yield rate and selectivity.

To deeply decode the reason why Pd‐CuCo_2_O_4_ catalyst achieves the excellent NO_3_RR performance, a series of investigations were further conducted. Firstly, it is widely recognized that NO_2_^−^ is a vital intermediate during NO_3_RR and the deeper reduction of NO_2_^−^ to NH_3_ is of equal importance [35, 36]. Therefore, the relay catalysis mechanism on Cu─Co dual sites was studied in the presence of Pd. As shown in Figure 3a, the FE of NO_2_^−^ (FE_NO2−_) for the Pd doped Cu/Co‐based catalysts were calculated based on UV–Vis method (Figures S18 and S19). It is worth noting that Pd‐Co_3_O_4_ exhibits the lowest FE_NO2−_ at the same potential while Pd‐CuO*_x_* shows the highest one, manifesting that Co is the main active site for NO_2_^−^ reduction to NH_3_. In addition, the FE_NO2−_ of Pd‐CuCo_2_O_4_ could be significantly reduced compared with Pd‐CuO*_x_*. Therefore, it is believed that the Cu sites could effectively reduce NO_3_^−^ to NO_2_^−^, while Co‐based component is critical for the further reduction of NO_2_^−^ to NH_3_. To further elucidate the relay catalysis mechanism in the catalysts, we investigated the rate constants of NO_3_^−^‐to‐NO_2_^−^ (k_1_) and NO_2_^−^‐to‐NH_3_ (k_2_) based on UV–Vis method (Figure S20) [37]. As shown in Figure 3b, Pd‐CuO*_x_* achieves the k_1_ of 10.40 × 10^−3^ min^−1^, which is higher than that of Pd‐Co_3_O_4_ (9.69×10^−3^ min^−1^), suggesting the faster reduction of NO_3_^−^‐to‐NO_2_^−^ in Cu‐based components. In contrast, a higher k_2_ is obtained on Pd‐Co_3_O_4_ (10.17 × 10^−3^ min^−1^) compared with Pd‐CuO*_x_* (5.47 × 10^−3^ min^−1^), showing the outperforming NO_2_^−^‐to‐NH_3_ ability in Co‐based catalyst. Therefore, it can be inferred that Cu and Co‐based component achieve the effect of relay catalysis during NO_3_RR. In addition, the Pd‐CuCo_2_O_4_ catalyst exhibits the highest k_1_ (11.64×10^−3^ min^−1^) and k_2_ (10.33×10^−3^ min^−1^) among the three catalysts, highlighting the synergistic relay catalytic effect between Cu─Co based component for accelerating the reaction kinetics.

**FIGURE 3:**
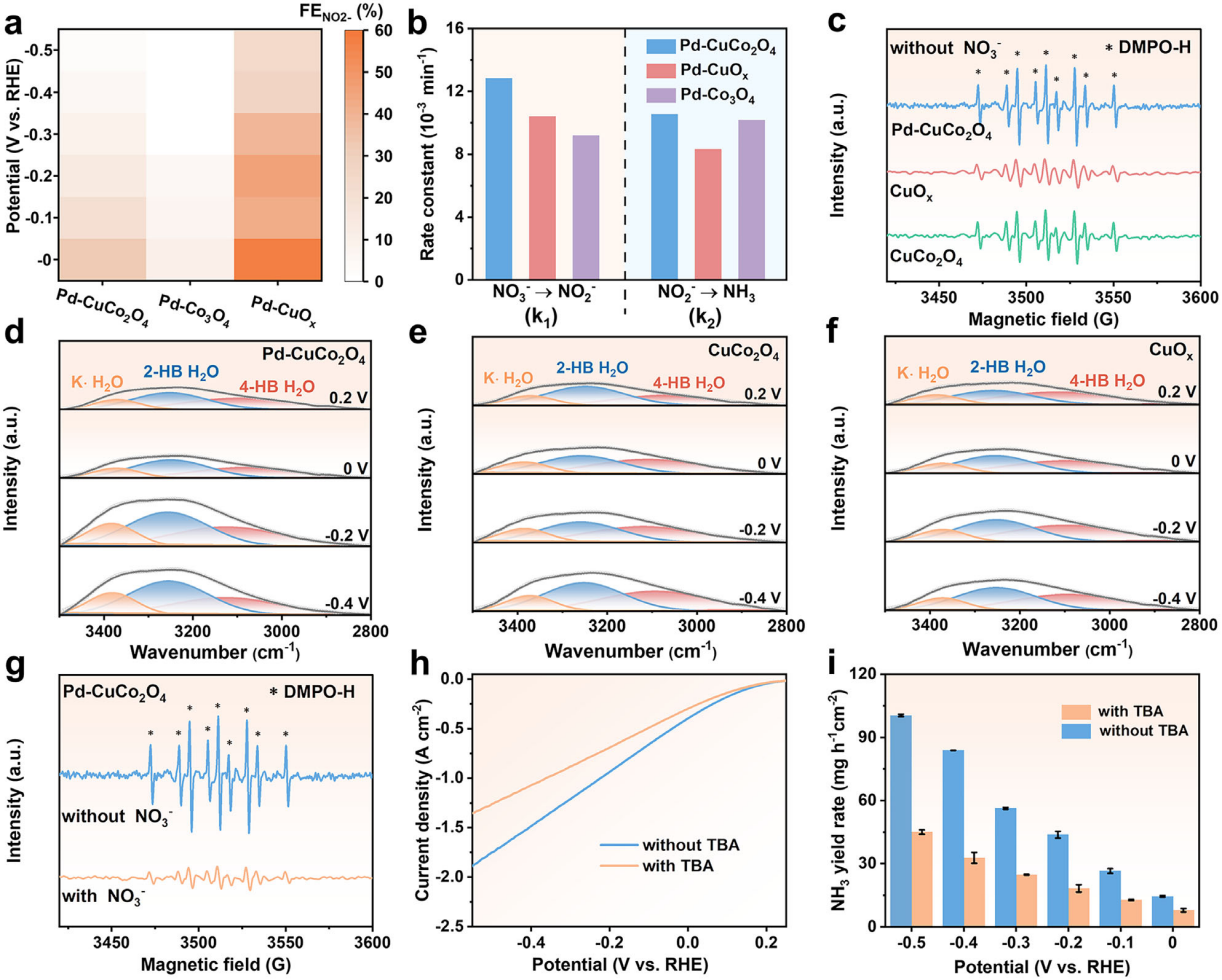
(a) FE of NO_2_^−^ for Pd‐CuCo_2_O_4_, Pd‐Co_3_O_4_ and Pd‐CuO*_x_* catalysts at different potentials. (b) Reaction rate constants of Pd‐CuCo_2_O_4_, Pd‐CuO*_x_* and Pd‐Co_3_O_4_ catalysts in NO_3_RR and NO_2_RR processes. (c) EPR spectra obtained by electrolysis of Pd‐CuCo_2_O_4_, CuO*_x_* and CuCo_2_O_4_ catalysts in 1.0 m KOH solution using DMPO as the *H trapping reagent. In situ ATR‐FTIR spectra of interfacial H_2_O on (d) Pd‐CuCo_2_O_4_, (e) CuCo_2_O_4_ and (f) CuO*_x_* at different potentials. (g) EPR spectra obtained by electrolysis of Pd‐CuCo_2_O_4_ in 1.0 m KOH solution with and without NO_3_^−^ in 1.0 m KOH solution using DMPO as the *H trapping reagent. (h) LSV curves of Pd‐CuCo_2_O_4_ catalyst with and without TBA. (i) The NH_3_ yield rate of Pd‐CuCo_2_O_4_ catalyst with and without TBA at different potentials.

Despite the construction of Cu─Co dual sites for relay catalysis, the supply of *H for hydrogenation steps is no less significantly crucial in enabling the production of NH_3_. The generated *H in this work was confirmed by the electron paramagnetic resonance (EPR) spectra using 5,5‐dimethyl‐1‐pyrroline N‐oxide (DMPO) as the *H trapping reagent. As compared in Figure 3c, the EPR spectra of the as‐prepared catalysts tested in 1.0 m KOH displays distinct 9 signals with the intensity ratio of 1:1:2:1:2:1:2:1:1, which can be assigned to the spin adduct of DMPO‐H [38, 39]. Notably, CuO*_x_* shows weak DMPO‐H signals while the signal intensity enhanced under CuCo_2_O_4_ catalyst, suggesting that Co acts as the main active center for *H generation instead of Cu. Moreover, the DMPO‐H signal further improved significantly compared to CuCo_2_O_4_, manifesting the importance of Pd doping effect for *H supply. In light of this, it can be concluded that the introduction of Pd effectively promotes the *H generation, which is the key promoter for *H‐NO*_x_* coupling on Co sites. On this basis, in situ attenuated total reflection Fourier‐transform infrared spectroscopy (in situ ATR‐FTIR) was conducted to investigate the regulation of interfacial H_2_O on different active sites. As shown in Figure 3d–f, all catalysts exhibit a broad peak ranging from 2800 to 3600 cm^−1^, which is assigned to the O─H stretching mode of H_2_O [40]. The peak of interfacial H_2_O can be deconvoluted into 4‐coordinated hydrogen‐bonded H_2_O (4‐HB H_2_O), 2‐coordinated hydrogen‐bonded H_2_O (2‐HB H_2_O) and K^+^‐ion hydrated H_2_O (K^+^·H_2_O). It is widely recognized that the K^+^·H_2_O species with a weak hydrogen‐bonded structure is beneficial for *H generation through H_2_O dissociation [40]. Therefore, the content of K^+^·H_2_O was further determined with the potential negatively shifted from +0.2 to −0.4 V (vs RHE). It is evident that the K^+^·H_2_O peak intensity gradually increases as the potential shifts negatively in Pd‐CuCo_2_O_4_, suggesting the enhanced H_2_O dissociation ability under negative potentials (Figure 3d). Of note, the peak intensity of K^+^·H_2_O on Pd‐CuCo_2_O_4_ is much stronger than those of CuCo_2_O_4_ and CuO*_x_* under the same potential (Figure 3d–f), indicating that the Pd doping effect effectively optimized the interfacial H_2_O structure on Co sites to form active K^+^·H_2_O, thereby promoting H_2_O dissociation and *H supply. Further, EPR spectrum after adding NO_3_^−^ during electrolysis was also conducted and the significantly weakened peaks irrefutably confirming the participation of *H in hydrogenation process in NO_3_RR (Figure 3g). On this basis, hydroxyl radical scavenger tert‐butanol (TBA) was also applied as the *H trapping reagent and NO_3_RR were conducted with/without TBA under Pd‐CuCo_2_O_4_ catalyst. The distinct decrease in NO_3_RR current density (Figure 3h and Figure S21) and NH_3_ yield rates (Figure 3i) upon the addition of TBA confirms the involvement of *H in the protonation process, which is consistent with the EPR results.

### 2.3 Mechanism Insight

To deeply unravel the underlying reaction mechanism of NO_3_RR over Pd‐CuCo_2_O_4_ catalyst, insitu ATR‑FTIR tests under different potentials were applied to probe the intermediates during NO_3_RR. As depicted in Figure 4a, the characteristic vibrational peaks for adsorbed *NO_3_^−^ (1355 cm^−1^) and *NO_2_^−^ (1282 cm^−1^) are detectable, verifying the adsorption and initial reduction of nitrate species on the catalyst surface. A set of hydrogen‐containing signals, including NH_2_OH (1075 cm^−1^), *NH_2_ (1170 cm^−1^), N─H vibration (1421 cm^−1^), and NH_4_^+^ (1477 cm^−1^), grow progressively more intense as the applied potential moves negatively, confirming effective hydrogenation of *NO*_x_* intermediates. The broad peak intensity at 1631 cm^−1^ corresponding to *NO and H_2_O also increases at more negative potentials, revealing H_2_O dissociation can be facilitated to supply *H species for *H─NO*_x_* coupling. Density of states (DOS) analysis further discloses the electronic origin for improved NO_3_^−^ adsorption. For pristine CuCo_2_O_4_, the d‐band centers of Cu and Co are located at −2.25 and −1.81 eV, respectively (Figure 4b), which are closer to the Fermi level relative to CuO (−2.42 eV) and Co_3_O_4_ (−1.86 eV) (Figure S22), suggesting that the formation of Cu─Co synergistic sites in CuCo_2_O_4_ induces an upshift of their d‐band centers. Upon Pd doping, the d‐band center of Cu shifts distinctly upward to −1.65 eV, strengthening orbital coupling between Cu 3d orbitals and adsorbed NO_3_^−^ orbitals and facilitating NO_3_^−^ adsorption during the initial NO_3_RR pathway.

**FIGURE 4:**
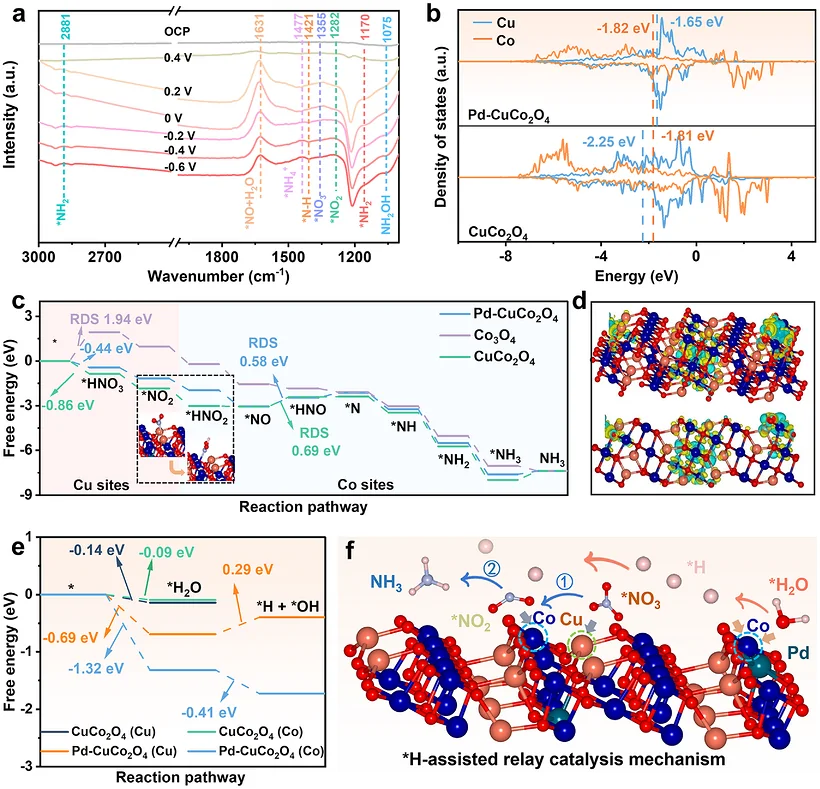
(a) In situ ATR‑FTIR for Pd‐CuCo_2_O_4_ during NO_3_RR under different potentials. (b) DOS analysis of Pd‐CuCo_2_O_4_(311) and CuCo_2_O_4_(311). (c) The Gibbs free energy change of NO_3_RR on Pd‐CuCo_2_O_4_(311), CuCo_2_O_4_(311) and Co_3_O_4_(311). (d) Charge density difference of Pd‐CuCo_2_O_4_(311) where the yellow cloud represents charge accumulation while the cyan cloud represents charge dissipation. Color code: dark green, Pd; orange, Cu; dark blue, Co; red, O. (e) The Gibbs free energy change of H_2_O adsorption on Pd‐CuCo_2_O_4_(311) and CuCo_2_O_4_(311) as well as the *H generation on Pd‐CuCo_2_O_4_(311). (f) Schematic illustration of the proposed *H‐assisted relay catalysis during NO_3_RR process on the Pd‐CuCo_2_O_4_(311).

On this basis, the characteristics of *H‐assisted relay catalysis mechanism of Pd‐CuCo_2_O_4_ during NO_3_RR was further elucidated [41]. In order to clarify the effect of constructing Cu─Co dual site and the Pd doping effect, CuCo_2_O_4_(311) and Co_3_O_4_(311) were selected as the control groups for Pd‐CuCo_2_O_4_(311). As depicted in Figure 4c, the reaction pathway for NO_3_RR is as follows: *HNO_3_ → *NO_2_ → *HNO_2_ →*NO → *HNO → *N → *NH →*NH_2_ → *NH_3_ and the schematic of intermediate structures on Pd‐CuCo_2_O_4_(311), CuCo_2_O_4_(311) and Co_3_O_4_(311) surfaces are shown in Figures S23–S25, respectively. Initially, NO_3_^−^ hydrogenation is prone to occur on the Cu site of CuCo_2_O_4_ with a free energy change (Δ*G*) of −0.86 eV, while this step is endothermic with the ΔG of 1.94 eV on Co_3_O_4_, indicating the adsorption for HNO_3_ is inhibited on Co sites and highlighting the importance of substituting partial Co atoms with Cu to construct CuCo_2_O_4_. Interestingly, adsorption site is transferred from Cu to Co during the hydrogenation step of *NO_2_ → *HNO_2_ and the relay catalysis is realized on CuCo_2_O_4_ component in both of the Pd‐CuCo_2_O_4_(311) and CuCo_2_O_4_(311), as shown in the inset configurations of Figure 4c. In addition, the generation of *HNO serves as the rate‐determining step (RDS) of NO_3_RR on Pd‐CuCo_2_O_4_(311) with the Δ*G* of 0.58 eV, which is lower than that of CuCo_2_O_4_(311) (0.69 eV), suggesting that Pd doping effect could further facilitate NO_3_RR by lowering the RDS energy barrier. On the other hand, the incorporation of Pd dopants into CuCo_2_O_4_ modulates the electronic structure of Cu and Co sites (Figure 4d) and thus promotes the *H supply. As shown in Figure 4e, the Δ*G* for the H_2_O adsorption on Cu site and Co site of CuCo_2_O_4_ is −0.14 and −0.09 eV, and reduced to −0.69 and −1.32 eV respectively after Pd incorporation, indicating the significant enhancement for H_2_O adsorption through Pd doping strategy. Therefore, Co sites are considered as the H_2_O adsorption sites due to the lower Δ*G*. It should be noted that an uphill Δ*G* of 0.29 eV is needed for *H generation on Cu sites while this step is exothermic on Co sites (−0.41 eV). Therefore, it can be speculated that *H supply is realized on Pd modified Co sites, which is in line with the results of EPR spectra. After which the co‐adsorption for the *H and *NO_2_ could be accomplished on bi‐functional Co sites and efficient NH_3_ production could be realized based on the *H‐assisted NO_3_^–^ → NO_2_^–^ → NH_3_ relay catalysis. Based on the above DFT calculations and experimental evidence, it can be inferred that the NO_3_^–^ → NO_2_^–^ → NH_3_ relay catalysis occurs on the Cu─Co pair. Of particular note, the *H spillover on the Pd‐modified Co sites overcomes the *H and *NO_2_ coupling bottleneck, which is the decisive factor behind the outstanding NH_3_ production performance (Figure 4f).

### 2.4 Practical Applications

As the Pd‐CuCo_2_O_4_ catalyst exhibits excellent NO_3_RR performances due to the *H‐assisted relay catalysis process, the practical applications abilities were further evaluated. As shown in Figure 5a, time‐dependent concentration profiles of NO_3_^−^ reagent, NO_2_^−^ intermediate, and NH_3_ product were monitored to measure the NO_3_^−^ removal capacity of Pd‐CuCo_2_O_4_ catalyst at −0.4 V (vs RHE). Impressively, NO_3_^−^ was reduced to NH_3_ efficiently with the rapid decrease of NO_3_^−^─N and increase of NH_3_─N simultaneously. In addition, the NO_3_^−^─N concentration dropped below the World Health Organization (WHO) drinking water standard (10 mg L^−1^) [35] within 45 minutes and NO_3_^–^‐to‐NH_3_ conversion rate reaches 88.0% after 75 minutes, indicating efficient NO_3_^−^ removal ability in the wastewater treatment. Besides, the NO_2_^−^─N concentration consistently remains below 0.9 mg L^−1^ (WHO NO_2_^−^─N regulation) [35], suggesting that the NO_2_^−^ intermediate could be rapidly converted to NH_3_ with the help of *H‐assisted relay catalysis mechanism in Pd‐CuCo_2_O_4_ catalyst. No N_2_H_4_ can be detected by UV–Vis spectrophotometry measurements (Figure S26), further suggesting the excellent selectivity for NH_3_ synthesis. The influence of NO_3_^−^ concentration on the NO_3_RR performance was also investigated by chronoamperometry at −0.4 V (vs RHE) (Figure S27). As listed in Figure 5b, the NH_3_ yield rate increases obviously from 17.86 to 83.86 mg h^−1^ cm^−2^ as NO_3_^−^ concentration rises from 0.01 to 0.1 m, and then slightly decreases to 83.57 mg h^−1^ cm^−2^ with the further increase of NO_3_^−^ concentration to 0.15 m, which may result from the insufficient *H supply in this case. FE_NH3_ exhibits volcano shape with the increase of NO_3_^−^ concentration, peaking at 90.05% under 0.1 m NO_3_^−^. This trend suggests that the adsorption and conversion efficiency of NO_3_^−^ is optimized at 0.1 m NO_3_^−^, which can be attributed to the equilibrium established between the availability of *H and intermediates absorption sites under Pd‐CuCo_2_O_4_ catalyst. The durability of the Pd‐CuCo_2_O_4_ catalyst in NO_3_RR was also studied in this work (Figure S28). Both the NH_3_ yield rate and FE_NH3_ remain stable can be well maintained during 10 cycles (Figure 5c). The SEM image and XPS results after stability test confirm the rigidity of the microsphere morphology and chemical composition (Figure S29). Encouraged by the excellent catalytic activity of Pd‐CuCo_2_O_4_ catalyst, zinc‐nitrate (Zn‐NO_3_^−^) battery was assembled using Zn plate as the anode and Pd‐CuCo_2_O_4_ as the cathode, respectively [42, 43]. As shown in Figure 5d, the open‐circuit voltage (OCV) of the zinc–nitrate battery reaches to 1.443 V (vs Zn/Zn^2+^). The current density increases with the decrease of discharging potential and achieves the maximum current density of 86.38 mA cm^−2^. Moreover, Pd‐CuCo_2_O_4_‐based Zn‐NO_3_^−^ battery exhibits a maximum power density of 19.93 mW cm^−2^ at 42.88 mA cm^−2^. A ladder‐shaped profile of voltage with stable discharge plateaus are observed when the battery is discharged at different current densities, indicating favorable discharge stability (Figure 5e). Based on the discharge current density, NH_3_ yield rates were obtained (Figure S30), further confirming the practical potential of Zn─NO_3_^−^ battery.

**FIGURE 5:**
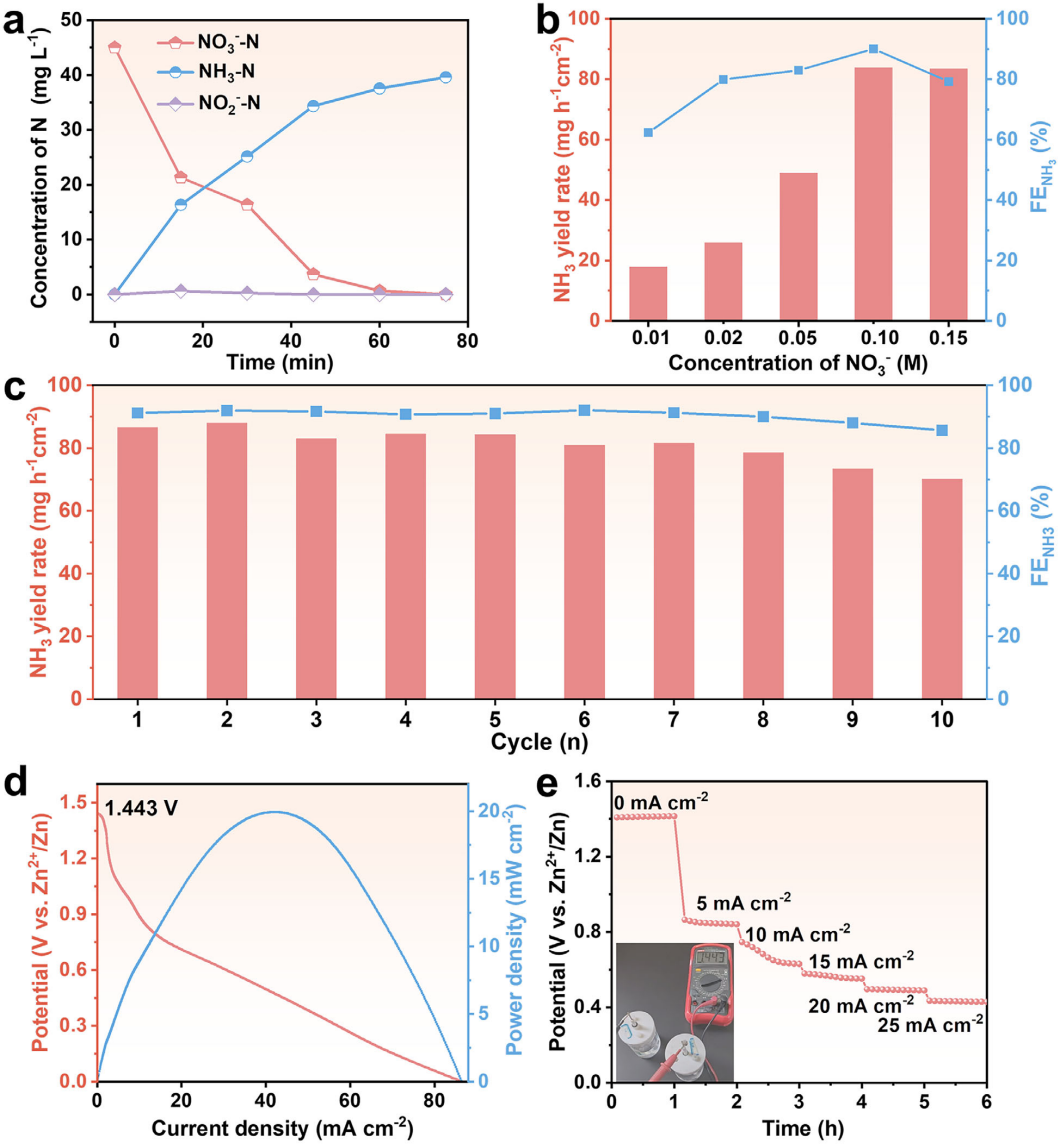
(a) Time‐dependent concentration changes of NO_3_^−^─N, NH_3_─N and NO_2_^−^─N during NO_3_RR. (b) NO_3_RR performance at −0.4 V (vs RHE) under different NO_3_^−^ concentrations. (c) Cyclic stability test of NO_3_RR at −0.4 V (vs RHE). (d) Discharge polarization curve and power density of Zn─NO_3_^−^ battery. (e) Galvanostatic discharge curves at different current densities.

## 3 Conclusion

In conclusion, a robust hollow spiny microsphere Pd‐CuCo_2_O_4_ catalyst is explored based on the increasing demand for high NH_3_ yield under ampere‐level current densities during NO_3_RR. The superior properties for NH_3_ synthesis stem from its relay catalysis mechanism which promotes the NO_3_^−^ to NO_2_^−^ and NO_2_^−^ to NH_3_ on Pd modified Cu─Co relay sites. Moreover, *H generation is facilitated by Pd doping effect, which participates in the hydrogenation process, effectively eliminating the *H‐NO*_x_* coupling bottleneck and ensuring the smooth progression of the relay catalysis. Combining the advantages of morphology and Pd doped Cu─Co relay active sites, Pd‐CuCo_2_O_4_ catalyst achieves an ultra‐high current density over −2.0 A cm^−2^ with the maximum NH_3_ yield rate of 100.43 mg h^−1^ cm^−2^ in 0.1 m KNO_3_. This work opens an avenue for catalyst design and provides possibility in Zn─NO_3_^−^ battery, which is feasible for practical applications.

## 4 Experimental Section

### 4.1 Preparation of Catalysts

The Pd‐CuCo_2_O_4_ catalyst was synthesized through a hydrothermal method followed by a calcination process. As a typical demonstration, 1 mmol Cu(NO_3_)_2_·3H_2_O, 2 mmol Co(NO_3_)_2_·6H_2_O, 5 mmol urea, 5 mmol NH_4_F and 1 mL, 0.1 mm Na_2_PdCl_4_ were dissolved in 35 mL deionized water to form a transparent pink solution. After immersing a piece of nickel (Ni) foam in the above solution, the mixture was transferred into a Teflon‐lined stainless‐steel autoclave and the hydrothermal process was conducted at 120°C for 4 h. After natural cooling to room temperature, the obtained precursor was washed with water and ethanol. After that, precursor was calcined at 400°C for 2 h with a heating rate of 5°C min^−1^ in air to obtain the Pd‐CuCo_2_O_4_ catalyst. The preparation process of CuCo_2_O_4_ is similar to the Pd‐CuCo_2_O_4_ except that Na_2_PdCl_4_ was absent during the solution preparation process. The preparation process of Pd‐CuO*_x_*, Pd‐Co_3_O_4_ counterparts are also similar to Pd‐CuCo_2_O_4_ except by replacing 1 mmol Cu(NO_3_)_2_·3H_2_O and 2 mmol Co(NO_3_)_2_·6H_2_O with 3 mmol Cu(NO_3_)_2_·3H_2_O, 3 mmol Co(NO_3_)_2_·6H_2_O, respectively. L‐Pd‐CuCo_2_O_4_ and H‐Pd‐CuCo_2_O_4_ were synthesized by adjusting the Na_2_PdCl_4_ dosage to 0.5 mL and 1.5 mL, respectively.

### 4.2 Electrochemical Experiments

All electrochemical tests were carried out using a GAMRY electrochemical workstation. The cathode and anode chamber were separated by a Nafion 211 proton exchange membrane. In a three‐electrode system, the as‐synthesized catalysts, Hg/HgO electrode, and Pt tablets were designated as working, reference, and counter electrodes, respectively. All potentials above were converted into reversible hydrogen electrode (RHE) by the following equation: *E*(vs RHE) = *E*(vs Hg/HgO) + 0.098 + 0.059 × pH. The volume of the electrolyte is 60 mL in each chamber and the surface area of the working electrode was controlled at 0.25 cm^2^. Linear sweep voltammetry (LSV) was performed at a scan rate of 10 mV s^−1^ in a potential range from 0 to −0.5 V (vs RHE) in a 1.0 m KOH solution. Electrochemical impedance spectroscopy (EIS) measurements were executed by applying a sinusoidal waveform across a frequency range of 0.01 Hz to 100 kHz. Electrochemically active surface area (ECSA) was measured by cyclic voltammetry (CV) at different scan rates of 20, 40, 60, 80, and 100 mV s^−1^. The rate constant (k) was calculated by monitoring the NO_3_^−^ or NO_2_^−^ concentrations during NO_3_^−^ reduction and NO_2_^−^ reduction, respectively. It assumes that the concentration of NO_3_^−^ or NO_2_^−^ decays exponentially at a first‐order rate. The values of k_1_ and k_2_ were calculated according to the following formula: *C*_t_ = *C*_0_ × exp(−k × t), where *C*_0_ is the initial NO_3_^−^ or NO_2_^−^ concentration and *C*_t_ is the NO_3_^−^ or NO_2_^−^ concentration of −0.4 V (vs RHE) at reaction time.

## Author Contributions

**Yuhuan Cui**: conceptualization, Writing – review and editing, funding acquisition, Writing – original draft, methodology. **Yingying Xu**: data curation, investigation, validation. **Jingyao Guo**: formal analysis, visualization. **Yao Liu**: investigation, visualization. **Yuxuan Li**: validation, writing – review and editing. **Zixuan Feng**: data curation. **Yitong Zhou**: software, formal analysis. **Yongjun Gao**: writing – review and editing, funding acquisition, resources. **Zhili Wang**: supervision, writing – review and editing, methodology.

## Funding

This work was supported by the National Science Foundation of Hebei Province (E2024201032), Hebei Province Innovation Capability Enhancement Plan Project (22567620H), National Natural Science Foundation of China (22378094), and Research Initiation Program of Hebei University (521100223242).

## Conflicts of Interest

The authors declare no conflicts of interest.

## Supporting information




**Supporting File**: advs78576‐sup‐0001‐SuppMat.docx.

## Data Availability

The raw experimental and theoretical datasets that support the results presented in this work are available from the corresponding author upon reasonable request.
