# Supplementary material for: Overcoming the *H‐NO x Coupling Bottleneck via Palladium‐Enabled Relay Catalysis on CuCo2O4 Toward Ampere‐Level Nitrate‐to‐Ammonia
Source: Adv Sci (Weinh). 2026 Oct 29:e78576. Online ahead of print. doi: 10.1002/advs.78576 (PMC13720387; doi:10.1002/advs.78576)
Supplement: Supplementary file 1 [file ADVS-9999-e78576-s001.docx]

Supporting Information

**Overcoming the *H-NO_x_ Coupling Bottleneck via Palladium-Enabled Relay Catalysis on CuCo_2_O_4_ toward Ampere-Level Nitrate-to-Ammonia**

*Yuhuan Cui*^a^, Yingying Xu^a^, Jingyao Guo^a^, Yao Liu^a^, Yuxuan Li^a^, Zixuan Feng^b^, Yitong Zhou^c^, Yongjun Gao*^a^, and Zhili Wang*^b^*

1. **Chemicals and materials**

Salicylic acid (C_7_H_6_O_3_, 99.5%), trisodium citrate dihydrate (C_6_H_5_Na_3_O_7_·2H_2_O, 99.0%), sodium hypochlorite solution (NaClO, effective chlorine: 7.5%), deuterium oxide (D_2_O, 99.9 atom%) ,^15^N labeled potassium nitrite (K^15^NO_3_, 99.0 atom%), Ammonium chloride (NH_4_Cl, 99.0%), cobalt nitrate hexahydrate (Co(NO_3_)_2_·6H_2_O, 99.0%), potassium hydroxide (KOH, 85%), sulfanilamide (C_6_H_8_O_2_N_2_S, 99.5%), trisodium citrate dihydrate (C_6_H_5_Na_3_O_7_·2H_2_O, 99.0%), naphthalene ethylenediamine hydrochloride (C_12_H_14_N_2_·2HCl, 98.0%), sulfamic acid (NH_2_SO_3_H, 99.5%), sodium nitroprusside (C_5_H_4_FeN_6_Na_2_O_3_, 99.0%), urea (CH_4_N_2_O, 99.0%), tert-Butanol (C_4_H_10_O, 99.0%) and dimethylsulfoxide-d6 (DMSO-d6, D. 99.9% + 0.03% TMS) were purchased from Macklin reagent. Copper nitrate trihydrate (Cu(NO_3_)_2_·3H_2_O, 99.0%) was purchased from Sinopharm Chemical Reagent Co., Ltd. P-dimethylaminobenzaldehyde ((CH_3_)_2_NC_6_H_4_CHO, 98%) and ammonium fluoride (NH_4_F, 99.0%) were purchased from Tianjin Kemiou Chemical Reagent Co., Ltd. Palladium chloride (PdCl_2_, Pd 59.0%) was purchased from J&K Scientific. Ammonium chloride (NH_4_Cl, 99.5%) was purchased from Aladdin reagent. Ltd. N211 proton exchange membrane (N211) was purchased from Suzhou Sinero Technology Co., Ltd. ^14^N labeled potassium nitrite (KNO_3_, 99.0 atom%), hydrochloric acid (HCl, 36.0 ~ 38.0%), nitric acid (HNO_3_, 65.0 ~ 68.0%) and acetone (C_3_H_6_O, 99.0%) were purchased from Tianjin Damao Chemical Reagent. Phosphoric acid (H_3_PO_4_, 85.0%) and ethanol absolute (C_2_H_6_O, 99.7%) were purchased from Tianjin Huihang reagent.

**2. Characterizations**

Morphology and compositions of catalysts were characterized by field-emission scanning electron microscope (SEM, JEOL, JSM-7900F) equipped with energy dispersive spectroscopy (EDS). The electron micrographic structures were characterized by a field-emission transmission electron microscope (JEM 2100F, 200 kV). X-ray diffraction (XRD) was performed on a smartlab-9kw diffractometer using Cu-K_α_ radiation. The surface chemical analysis by X-ray photoelectron spectrometer (XPS) was performed using an ESCALAB 250 Xi system. The ^1^H nuclear magnetic resonance measurement were recorded on BUXI-I NMR. EPR spectra were performed on A300-10/12 from Bruker in Germany. Raman spectra were measured on a Raman spectrometer (LabRAM HR Evolution) equipped with 532 nm-wavelength lasers as the excitation source. The ultraviolet-visible (UV-Vis) absorbance spectra were obtained by Beijing Puxi spectrophotometer. X-ray absorption spectroscopy (XAS) was tested using the BL14W1 beamline at the Shanghai Synchrotron Radiation Facility (SSRF, China). The in-situ attenuated total reflection surface-enhanced infrared absorption spectroscopy (ATR-SEIRAS) measurements were carried out on a Thermo Scientific Nicolet 6700 Fourier transform infrared (FTIR) spectrometer equipped with OMNIC software and a liquid-nitrogen-cooled mercury cadmium telluride (MCT) detector.

**3. Quantification of NH_3_**

The indophenol blue method was used to test the concentration of NH_3_. After electrolysis, a certain amount of electrolyte was diluted to 2.0 mL with 1.0 m KOH. Then, 2.0 mL NaOH (1.0 m) containing 5 wt% C_7_H_6_O_3_ and C_6_H_5_Na_3_O_7_·2H_2_O, 1.0 mL, 0.05 m NaClO, and 0.2 mL 1 wt% C_5_FeN_6_Na_2_O∙2H_2_O were added in turn into the electrolyte and kept at room temperature for 2 hours. Then, the mixture solution was tested using a UV-Vis spectrophotometer to determine the absorbance of NH_4_^+^. The concentration of NH_3_ is determined by absorbance at ~650 nm. Calibration curves were constructed using standard NH_4_Cl solutions[1].

The yield of NH_3_ was calculated by the following equation：

$\text{Y}_{{\text{N}\text{H}}_{\text{3}}}\text{=}\frac{\text{C}_{{\text{N}\text{H}}_{\text{3}}}\text{ }\text{× V}}{\text{S}\text{ }\text{×}\text{ }\text{t}}$	(1)

The FE of NH_3_ was calculated by the following equation：

$\text{FE}_{{\text{N}\text{H}}_{\text{3}}}\text{=}\frac{\text{8×F×C}_{{\text{N}\text{H}}_{\text{3}}}\text{ }\text{× V}}{\text{M}_{{\text{N}\text{H}}_{\text{3}}}\text{×Q}}$	(2)

where C_NH3_ is the concentration of NH_3_ (μg mL^−1^), V is the volume of the electrolyte (mL), S is the test area of the catalyst (cm^2^), t is the reaction time (h), Q is the total charge (C), F denotes the Faradaic constant (96485 C mol^−1^), M_NH3_ represents the molar mass of NH_3_ (17 g mol^−1^).

4. **Quantification of NO_2_^‒^**

The NO_2_^‒^ color agent was prepared by mixing 0.04 g C_12_H_14_N_2_·2HCl, 0.8 g C_6_H_8_O_2_N_2_S and 2.0 mL H_3_PO_4_ (ρ = 1.70 g mL^−1^) solution in 10.0 mL H_2_O. Then the electrolyte was diluted to 5.0 mL, 0.1 mL of the above color agent was added, and left at room temperature for 20 min. The NO_2_^‒^ concentration was measured using the UV-Vis spectrophotometer absorbance at the wavelength of 540 nm. The calibration curves were calibrated using a standard KNO_2_ solution with various concentrations[2].

The FE of NO_2_^‒^ was calculated by the following equation：

$\text{FE}_{\text{NO}_{\text{2}}^{\text{‒}}}\text{=}\frac{{\text{2}\text{×F×C}}_{{\text{N}\text{O}}_{\text{2}}^{\text{‒}}}\text{ }\text{× V}}{\text{M}_{\text{NO}_{\text{2}}^{\text{‒}}}\text{×Q}}$	(3)

where C is the concentration of NO_2_^‒^ (μg mL^−1^), V is the volume of the electrolyte (mL), S is the test area of the catalyst (cm^2^), t is the reaction time (h), Q is the total charge (C), F denotes the Faradaic constant (96485 C mol^−1^), M represents the molar mass of NO_2_^‒^ (46 g mol^−1^).

**5. Quantification of NO_3_^‒^**

The NO_3_^‒^ color agents consist of 0.8 wt% H_3_NO_3_S solution and 1.0 m HCl. After electrocatalysis, a certain amount of electrolytic mass was diluted to 5.0 mL. Then, 0.1 mL 1.0 m HCl solution and 0.01 mL 0.8 wt% H_3_NO_3_S were added to the above solution and left at room temperature for 20 min. The NO_3_^‒^ concentration was measured using the UV-Vis absorbance at the wavelength of 220 nm and 275 nm (A = A_220 nm_ ‒ 2 × A_275_ _nm_) with the calibration curve. The calibration curves were calibrated using a standard KNO_3_ solution with various concentrations[3].

**6. Quantification of N_2_H_4_**

The N_2_H_4_ color agent was prepared by mixing 0.8985 g C_9_H_11_NO, 4.5 mL HCl (36.0~38.0%) solution and 45 mL C_2_H_6_O. Then 2.0 mL of the above color agent was added to the 2.0 mL electrolyte and left at room temperature for 20 min. N_2_H_4_ concentration was measured using UV-Vis absorbance at wavelength calibration curve of 455 nm[4].

7. **Computational details**

All the spin-unrestricted density functional theory (DFT) + U calculations were performed using Vienna Ab-initio Simulation Package (VASP)[5, 6]. The exchange and correlation potential were determined with the Perdew, Burke, and Ernzerhof within the generalized gradient approximation (PBE-GGA) functional[7]. The interaction between electrons and ions were described by using projector augmented wave (PAW) method. A thermal smearing of 0.05 eV to the orbital occupation was applied to speed up electronic convergence. The Brillouin zone sampling was performed on meshes with a k-point spacing of 0.04 Å^‒1^[8]. The convergence tolerance of electronic structure and geometry optimization was 1×10^‒5^ eV, 0.02 eV/Å, respectively. The plane wave cut-off was set to 400 eV. In the DFT + U method, we picked up the effective U values (4.0  eV for Co) to treat the localized nature of Co 3d electrons, which can describe on-site Coulomb and exchange interaction of localized electrons. A vacuum space larger than 15 Å was used to prevent artificial interaction effect between the slab and their mirror images. The geometrical configurations and charge density difference plots were illustrated with VESTA software[9].

**Figure S1.** Synthetic routes of Pd-CuO_x_, Pd-CuCo_2_O_4_ and Pd-Co_3_O_4_ catalysts.

**Figure S2.** SEM image of (a) Pd-CuO_x_, (b) Pd-Co_3_O_4_ and (c) CuCo_2_O_4_ catalysts.

**Figure S3.** XRD pattern of Pd-CuO_x_, Pd-Co_3_O_4_ and CuCo_2_O_4_ catalysts.

**Figure S4.** (a) Cu LMM spectra of Pd-CuCo_2_O_4_ and CuCo_2_O_4_ catalyst. (b) O 1s and (c) Pd 3d high resolution XPS spectrum of Pd-CuCo_2_O_4_ catalyst.

**Figure S5.** Onset potentials for Pd-CuCo_2_O_4_, Pd-CuO_x_, Pd-Co_3_O_4_ and CuCo_2_O_4_ catalysts.

**Figure S6.** (a) I-t curves of Pd-CuCo_2_O_4_ catalyst at different applied potentials for 1 h in 1.0 m KOH and 0.1 m KNO_3_ and (b) corresponding UV-Vis absorption curves.

**Figure S7.** (a) I-t curves of Pd-Co_3_O_4_ catalyst at different applied potentials for 1 h in 1.0 m KOH and 0.1 m KNO_3_ and (b) corresponding UV-Vis absorption curves.

**Figure S8.** (a) I-t curves of Pd-CuO_x_ catalyst at different applied potentials for 1 h in 1.0 m KOH and 0.1 m KNO_3_ and (b) corresponding UV-Vis absorption curves.

**Figure S9.** (a) I-t curves of CuCo_2_O_4_ catalyst at different applied potentials for 1 h in 1.0 m KOH and 0.1 m KNO_3_ and (b) corresponding UV-Vis absorption curves.

**Figure S10.** (a) UV-Vis absorption spectra and (b) standard curves of NH_4_^+^ for different concentrations.

**Figure S11.** (a) ^1^H NMR spectra, (b) standard curves of ^14^NH_4_^+^ for different concentrations. (c) Quantification of NH_3_ via UV-Vis and NMR measurements at −0.4 V (vs. RHE).

**Figure S12.** CV curves of (a) Pd-CuCo_2_O_4_, (b) Pd-CuO_x_, (c) Pd-Co_3_O_4_ and (d) CuCo_2_O_4_ catalysts in 1.0 m KOH solution at different scanning rates.

**Figure S13.** The EIS Nyquist plots for Pd-CuCo_2_O_4_, CuCo_2_O_4_, Pd-CuO_x_ and Pd-Co_3_O_4_ catalysts.

**Figure S14.** SEM image of (a) L-Pd-CuCo_2_O_4_ and (b) H-Pd-CuCo_2_O_4_ catalysts.

**Figure S15.** XRD pattern of L-Pd-CuCo_2_O_4_ and H-Pd-CuCo_2_O_4_ catalysts.

**Figure S16.** (a) I-t curves of H-Pd-CuCo_2_O_4_ catalyst at different applied potentials for 1 h in 1.0 m KOH and 0.1 m KNO_3_ and (b) corresponding UV-Vis absorption curves. (c) I-t curves of L-Pd-CuCo_2_O_4_ catalyst at different applied potentials for 1 h in 1.0 m KOH and 0.1 m KNO_3_ and (d) corresponding UV-Vis absorption curves. (e) NH_3_ yield rate and (f) FE_NH3_ of CuCo_2_O_4_, L-Pd-CuCo_2_O_4_, Pd-CuCo_2_O_4_ and H-Pd-CuCo_2_O_4_ catalysts at different potentials.

**Figure S17.** (a)Cu 2p and (b) Co 2p high resolution XPS spectra of CuCo_2_O_4_, L-Pd-CuCo_2_O_4_, Pd-CuCo_2_O_4_ and H-Pd-CuCo_2_O_4_ catalysts.

**Figure S18.** (a) UV-Vis absorption spectra and (b) standard curves of NO_2_^−^ for different concentrations.

**Figure S19.** UV-Vis absorption curves of NO_2_^−^ under (a) Pd-CuCo_2_O_4_, (b) Pd-Co_3_O_4_ and (c) Pd-CuO_x_ catalysts after electrocatalysis in 1.0 m KOH and 0.1 m KNO_3_.

**Figure S20.** (a) UV-Vis absorption spectra and (b) standard curves of NO_3_^−^ for different concentrations.

**Figure S21.** I-t curves of the Pd-CuCoO_x_ catalyst with TBA at different potentials.

**Figure S22.** DOS analysis on (a) CuO and (b) Co_3_O_4_.

**Figure S23.** The geometric structures of intermediates during NO_3_RR on Pd-CuCo_2_O_4_(311) surfaces. Color code: dark green, Pd; orange, Cu; dark blue, Co; light blue, N; red, O; white, H.

**Figure S24.** The geometric structures of intermediates during NO_3_RR on CuCo_2_O_4_(311) surfaces. Color code: orange, Cu; dark blue, Co; light blue, N; red, O; white, H.

**Figure S25.** The geometric structures of intermediates during NO_3_RR on Co_3_O_4_(311) surfaces. Color code: dark bule, Co; light blue, N; red, O; white, H.

**Figure S26.** UV-Vis absorption spectra of N_2_H_4_ at different potentials of Pd-CuCo_2_O_4_ catalyst.

**Figure S27.** (a) I-t curves and (b) corresponding UV-Vis absorption curves of the Pd-CuCo_2_O_4_ catalyst at different NO_3_^−^ concentrations.

**Figure S28.** (a) I-t curves of Pd-CuCo_2_O_4_ catalyst at −0.4 V (vs. RHE) for the stability test and (b) corresponding UV-Vis absorption curves.

**Figure S29.** (a) SEM image, XPS spectra of (b) Cu 2p, and (c) Co 2p of Pd-CuCo_2_O_4_ after stability test.

**Figure S30.** NH_3_ yield rates for Zn-NO_3_^−^ battery at various discharging current densities.

**Table S1.** The atomic ratio of Pd in the Pd-CuCo_2_O_4_ with different Pd contents.

|  | L-Pd-CuCo_2_O_4_ | Pd-CuCo_2_O_4_ | H-Pd-CuCo_2_O_4_ |
| --- | --- | --- | --- |
| Concentration | 38.5 µg L⁻¹ | 78.4 µg L⁻¹ | 130.5 µg L⁻¹ |
| Atomic ratio | 0.9 at% | 1.7 at% | 2.8 at% |

**Table S2.** Comparison of NO_3_RR electrocatalytic activity of Pd-CuCo_2_O_4_ catalyst with other electrocatalysts under ambient conditions.

| **Catalyst** | **Concentration of NO_3_****^−^ (mm)** | **NH_3_ yield rate**  **(mg h^−1^ cm^−2^)** | **FE_NH3_ (%)** | **Potential**  **(V vs. RHE)** | **Ref.** |
| --- | --- | --- | --- | --- | --- |
| **Pd-CuCo_2_O_4_** | **100** | **100.43** | **87.93** | **−0.50** | **This work** |
|  |  | **83.86** | **90.05** | **−0.40** |  |
| (Cu_0.6_Co_0.4_)Co_2_O_4_ | 100 | 18.50 | 96.5 | −0.45 | [10] |
| Cu_x_S-Co_0.5_ | 100 | 2.30 | 95.6 | −0.40 | [11] |
| o-CoP/C@ Cu_3_P/CF | 100 | 26.70 | 90.3 | −0.25 | [12] |
| Cu_3_(HITP)_2_/CF | 100 | 19.9 | 71.78 | −0.9 | [13] |
| In-Pd | 100 | 1.12 | 87.2 | −0.6 | [14] |
| Cu/Cu_2_O | 100 | 3.73 | 93.9 | −0.9 | [15] |
| CoMn_2_O_4_/NC | 100 | 1.04 | 92.4 | −0.7 | [16] |
| TpBpy-Cu-F | 100 | 2.72 | 82.6 | −0.746 | [17] |
| NF/Ni_3_N-Cu | 100 | 20.23 | 98.71 | −0.3 | [18] |
| CuNi-LDHs | 100 | 2.73 | 94.65 | −0.4 | [19] |
| Cu SCCs | 100 | 1.99 | 96 | −0.5 | [20] |
| Ni_2_P/CP | 100 | 15.4 | 97.4 | −0.2 | [21] |
| NiPr-TPA-COF | 300 | 2.50 | 90 | −1.18 | [22] |
| NiO_4_-CCP | 500 | 2.15 | 94.7 | −0.7 | [23] |
| Ni/Ni(OH)_2_ | 500 | 84.74 | 98.5 | −0.467 | [24] |
| CNS=CoP | 1000 | 52.59 | 90.5 | −1.03 | [25] |

**References**

1. Q. Yan, R. Zhao, H. Chen, et al., "Revealing the Tandem Behavior of Iron-Group/Copper Binary Catalysts in the Electroreduction of Nitrate to Ammonia," *Advanced Functional Materials* 35 (2025): 2425084, https://doi.org/10.1002/adfm.202425084.

2. X. Shi, M. Xie, K. Yang, et al., "Synergistic Effect of Ni/Ni(OH)_2_ Core-Shell Catalyst Boosts Tandem Nitrate Reduction for Ampere-Level Ammonia Production," *Angewandte Chemie International Edition* 63 (2024): e202406750, https://doi.org/10.1002/anie.202406750.

3. X. Zhou, W. Xu, Y. Liang, et al., "Dynamically Restructuring Nanoporous Cu-Co Electrocatalyst for Efficient Nitrate Electroreduction to Ammonia," *ACS Catalysis* 14 (2024): 12251, https://doi.org/10.1021/acscatal.4c03336.

4. F. Wang, J. Xiang, G. Zhang, et al., "Single-Atom Co Alloyed Ru for Electrocatalytic Nitrite Reduction to Ammonia," *Nano Research* 17 (2024): 3660, https://doi.org/10.1007/s12274-023-6261-2.

5. Kresse, Furthmuller, "Efficient Iterative Schemes for Ab Initio Total-Energy Calculations Using a Plane-Wave Basis Set," *Physical review. B, Condensed matter* 54 (1996): 11169, https://doi.org/10.1103/PhysRevB.54.11169.

6. M. Cococcioni, S. de Gironcoli, "Linear Response Approach to the Calculation of the Effective Interaction Parameters in the Lda+U Method," *Physical Review B* 71 (2005): 035105, https://doi.org/10.1103/PhysRevB.71.035105.

7. J. P. Perdew, K. Burke, M. Ernzerhof, "Generalized Gradient Approximation Made Simple," *Physical Review Letters* 77 (1996): 3865, https://doi.org/10.1103/PhysRevLett.77.3865.

8. Methfessel, Paxton, "High-Precision Sampling for Brillouin-Zone Integration in Metals," *Physical review. B, Condensed matter* 40 (1989): 3616, https://doi.org/10.1103/PhysRevB.40.3616.

9. K. Momma, F. Izumi, "Vesta 3 for Three-Dimensional Visualization of Crystal, Volumetric and Morphology Data," *Journal of Applied Crystallography* 44 (2011): 1272, https://doi.org/10.1107/S0021889811038970.

10. Q. Hu, S. Qi, Q. Huo, et al., "Designing Efficient Nitrate Reduction Electrocatalysts by Identifying and Optimizing Active Sites of Co-Based Spinels," *Journal of The American Chemical Society* 146 (2023): 2967, https://doi.org/10.1021/jacs.3c06904.

11. M. Zhang, Z. Zhang, S. Zhang, et al., "Efficient Electrochemical Nitrate Reduction to Ammonia Driven by a Few Nanometer-Confined Built-in Electric Field," *ACS Catalysis* 14 (2024): 10437, https://doi.org/10.1021/acscatal.4c02317.

12. Y. Li, Z. Lu, L. Zheng, et al., "The Synergistic Catalysis Effect on Electrochemical Nitrate Reduction at the Dual-Function Active Sites of the Heterostructure," *Energy & Environmental Science* 17 (2024): 4582, https://doi.org/10.1039/d4ee00784k.

13. W. Wang, B. Chen, J. Guo, et al., "Fabrication of a Novel Cu Based Conjugated Coordination Polymer for Effective Electroreduction of Nitrate to Ammonia and Zn-Nitrate Batteries," *Advanced Functional Materials* 35 (2025): 2501057, https://doi.org/10.1002/adfm.202501057.

14. M. Xie, S. Tang, Z. Li, et al., "Intermetallic Single-Atom Alloy In-Pd Bimetallene for Neutral Electrosynthesis of Ammonia from Nitrate," *Journal of the American Chemical Society* 145 (2023): 13957, https://doi.org/10.1021/jacs.3c03432.

15. N. Zhou, Z. Wang, N. Zhang, et al., "Potential-Induced Synthesis and Structural Identification of Oxide-Derived Cu Electrocatalysts for Selective Nitrate Reduction to Ammonia," *ACS Catalysis* 13 (2023): 7529, https://doi.org/10.1021/acscatal.3c01315.

16. Z. Niu, S. Fan, X. Li, et al., "Interfacial Engineering of CoMn_2_O_4_/NC Induced Electronic Delocalization Boosts Electrocatalytic Nitrogen Oxyanions Reduction to Ammonia," *Applied Catalysis B-Environment and Energy* 322 (2023): 122090, https://doi.org/10.1016/j.apcatb.2022.122090.

17. Y. Zhu, H. Duan, C. G. Gruber, et al., "Boosting Electrocatalytic Nitrate Reduction through Enhanced Mass Transfer in Cu-Bipyridine 2d Covalent Organic Framework Films," *Angewandte Chemie International Edition* 64 (2025): e20242182, https://doi.org/10.1002/anie.202421821.

18. X. Ouyang, W. Qiao, Y. Yang, et al., "Intensifying Interfacial Reverse Hydrogen Spillover for Boosted Electrocatalytic Nitrate Reduction to Ammonia," *Angewandte Chemie International Edition* 64 (2025): e202422585, https://doi.org/10.1002/anie.202422585.

19. H. Li, S. Li, R. Guan, et al., "Modulating the Surface Concentration and Lifetime of Active Hydrogen in Cu-Based Layered Double Hydroxides for Electrocatalytic Nitrate Reduction to Ammonia," *ACS Catalysis* 14 (2024): 12042, https://doi.org/10.1021/acscatal.4c03245.

20. Y. Liu, W. Qiu, P. Wang, et al., "Pyridine-N-Rich Cu Single-Atom Catalyst Boosts Nitrate Electroreduction to Ammonia," *Applied Catalysis B-Environment and Energy* 340 (2024): 123228, https://doi.org/10.1016/j.apcatb.2023.123228.

21. G. Huang, H. Liu, Y. Li, et al., "Anion-Regulated Active Nickel Site for Nitrate Reduction in Efficient Ammonia Electrosynthesis and Zn-Nitrate Battery," *Advanced Functional Materials* 35 (2025): 2500577, https://doi.org/10.1002/adfm.202500577.

22. F. Lv, M. Sun, Y. Hu, et al., "Near-Unity Electrochemical Conversion of Nitrate to Ammonia on Crystalline Nickel Porphyrin-Based Covalent Organic Frameworks," *Energy & Environmental Science* 16 (2023): 201, https://doi.org/10.1039/d2ee02647c.

23. Y. Zhang, H. Zheng, K. Zhou, et al., "Conjugated Coordination Polymer as a New Platform for Efficient and Selective Electroreduction of Nitrate into Ammonia," *Advanced Materials* 35 (2023): 2209855, https://doi.org/10.1002/adma.202209855.

24. X. Shi, M. Xie, K. Yang, et al., "Synergistic Effect of Ni/Ni(OH)_2_ Core-Shell Catalyst Boosts Tandem Nitrate Reduction for Ampere-Level Ammonia Production," *Angewandte Chemie International Edition* 63 (2024): e202406750, https://doi.org/10.1002/anie.202406750.

25. K. Fan, W. Xie, J. Li, et al., "Active Hydrogen Boosts Electrochemical Nitrate Reduction to Ammonia," *Nature Communications* 13 (2022): 7958, https://doi.org/10.1038/s41467-022-35664-w.
